# Robot-assisted thoracoscopic esophagectomy for gastrointestinal stromal tumor of the esophagus: A case report

**DOI:** 10.1016/j.ijscr.2021.106335

**Published:** 2021-08-26

**Authors:** Hiroyuki Yamamoto, Yuma Ebihara, Kimitaka Tanaka, Aya Matsui, Yoshitsugu Nakanishi, Toshimichi Asano, Takehiro Noji, Yo Kurashima, Soichi Murakami, Toru Nakamura, Takahiro Tsuchikawa, Keisuke Okamura, Toshiaki Shichinohe, Satoshi Hirano

**Affiliations:** Department of Gastroenterological Surgery II, Hokkaido University Faculty School of Medicine, North 15 West 7, Kita-ku, Sapporo 0608638, Hokkaido, Japan

**Keywords:** GIST, gastrointestinal stromal tumor, RAMIE, robotic-assisted minimally invasive esophagectomy, EUS, endoscopic ultrasonography, FNA, fine-needle aspiration, CT, computed tomography, ICS, intercostal space, MIE, minimally invasive esophagectomy, Esophageal gastrointestinal stromal tumor, Robotic surgery, Minimally invasive, Esophagectomy

## Abstract

**Introduction:**

A gastrointestinal stromal tumor (GIST) often arises in the stomach and small intestine, while esophageal GIST is rare. The first-choice treatment is surgical resection, but there is no standard technique. Herein, we describe our experience in the treatment of esophageal GIST and discuss the usefulness of robotic esophagectomy.

**Presentation of case:**

The patient was a 60-year-old woman, who was diagnosed with a 30 mm GIST in the middle thoracic esophagus. We underwent robot-assisted thoracoscopic esophagectomy in the prone position. The duration of the thoracoscopic part was 69 min and the total operation time was 319 min. Total blood loss was 135 ml. The patient's postoperative course was uneventful after surgery and the patient was discharged home in good condition on the 18th postoperative day.

**Discussion:**

The prognosis of esophageal GIST was less favorable compared with gastric GIST, and due to the anatomical peculiarities of the esophagus, which surgical procedure should be performed is still under debate. Robotic surgery has several technological advantages as it provides a three-dimensional view, ten times magnification, tremor control, and ambidexterity. Therefore, Robotic-assisted minimally invasive esophagectomy (RAMIE) allows achieving for safe R0 resection of esophageal GIST.

**Conclusion:**

RAMIE may be useful for esophageal GIST because it facilitates safe and minimally invasive surgery in a limited space of the thoracic cavity.

## Highlights

• There is no standard technique due to the anatomical peculiarities of the esophagus.
• We think McKeown's procedure in the prone position has some advantages from others.
• Robot surgery has a three-dimensional view, tremor control, and ambidexterity.
• RAMIE would be useful techniques in a limited space of the thoracic cavity.

## 1 Introduction

Esophageal GIST is extremely rare and accounts for less than 1% of all GIST [1] and the prognosis of esophageal GIST is less favorable compared with gastric GIST [2]. Generally, the first-choice treatment is surgical resection, but there is no standard technique due to the anatomical peculiarity of the esophagus. It is essential to ensure negative margins without damaging the capsule because of malignant potential, esophagectomy might be preferable for the goal to achieve R0 resection by highest radicality. We report a patient with a GIST of the esophagus which was performed robot-assisted thoracoscopic esophagectomy in the prone position. This work has been reported in line with the SCARE criteria [3].

## 2 Presentation of case

### 2.1 Patient

**Fig. 1:**
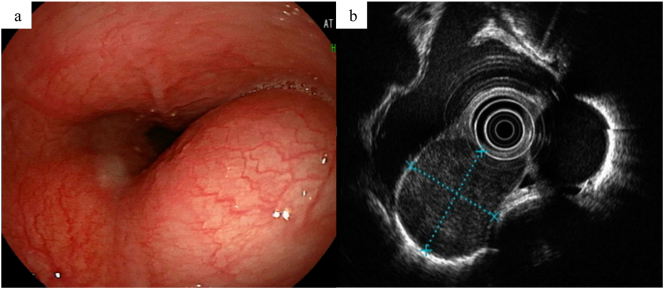
(a) Upper gastrointestinal endoscopy showed a protruded lesion covered with normal mucosa in 28–34 cm from the incisor teeth. (b) Subsequent EUS demonstrated a hypoechoic, homogeneous tumor 3.1 × 2.7 cm in size, arising from the muscular mucosae.

**Fig. 2:**
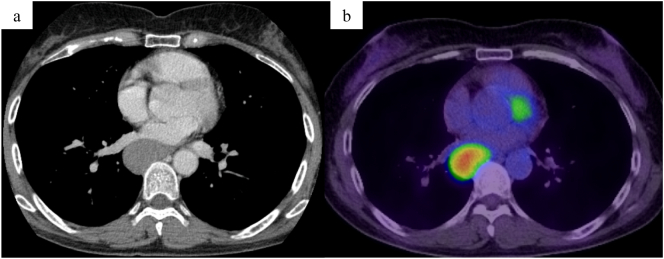
(a) CT scan showed the tumor with heterogeneous density and no metastasis in the lung and the liver. (b) Positron Emission Tomography CT revealed SUVmax 9.67 in the mediastinum on the right side of the esophagus.

A 60-year-old woman, complaining of abdominal pain, was monitored endoscopically for an esophageal submucosal tumor and referred to our hospital for evaluation. Her blood tests and physical examination revealed no abnormalities. Upper gastrointestinal endoscopy showed a protruded lesion covered with normal mucosa in 28–34 cm from the incisor teeth (Fig. 1a). Subsequent endoscopic ultrasonography (EUS) demonstrated a hypoechoic, homogeneous tumor 3.1 × 2.7 cm in size, arising from the muscular mucosae (Fig. 1b). A fine-needle aspiration (FNA) of the tumor showed a GIST. A computed tomography (CT) scan showed the tumor with heterogeneous density and no metastasis in the lung and the liver (Fig. 2a). Positron Emission Tomography CT revealed SUVmax 9.67 in the mediastinum on the right side of the esophagus (Fig. 2b).

### 2.2 Surgical technique

#### 2.2.1 Thoracoscopic surgery

**Fig. 3:**
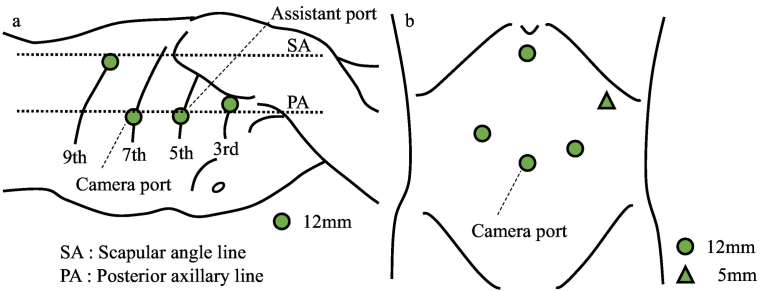
The port placement. (a) Thoracoscopic phase. (b) Laparoscopic phase.

**Fig. 4:**
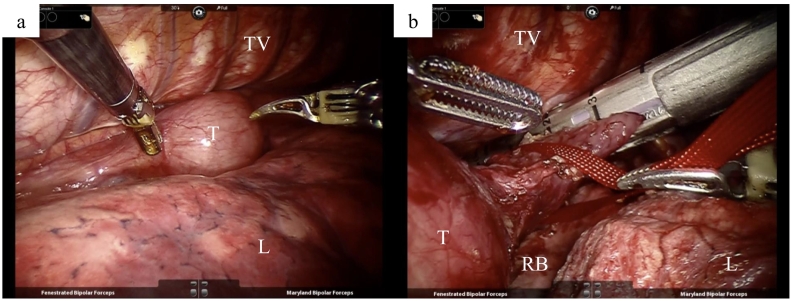
Intraoperative findings. (a) The operative field was ensured because the collapsed lung is drawn ventrally by gravity. (b) The esophagus was lifted by a vessel tape and divided at the tracheal bifurcation by the Stapler. T, tumor; L, lung; TV, thoracic vertebrae; RB, right main bronchus.

Under general anesthesia, the patient was intubated with a single lumen tube and placed in a prone position. The thoracoscopic phase was performed with the support of Da Vinci Si™ robotic system (Surgical Intuitive Inc., Mountain View, CA, USA). The port positions were as follows (Fig. 3a): The first port was carefully inserted into the 9th intercostal space (ICS) on the scapular angle line, and CO2 was then insufflated at a pressure of 8–10 mm Hg. The other three ports were under thoracoscopic control: a 12-mm port in the 3rd and 5th ICS behind the posterior axillary line and in the 7th ICS on a little ventral side of the posterior axillary line. After starting the artificial pneumothorax, the lung was gradually collapsed, and the operative field was ensured because the collapsed lung is drawn ventrally by gravity (Fig. 4a). Intrathoracic procedures were as follows: the right pulmonary ligament was divided, and the middle and lower esophagus was mobilized. The azygos vein was divided and the right bronchial artery was preserved. Then, the upper thoracic esophagus, the right main branch of the vagus nerve, and the right subclavian artery were exposed. Then, the upper thoracic esophagus was circumferentially mobilized and divided at the tracheal bifurcation by Stapler (Fig. 4b). There was no evidence of tumor invasion of other organs, and by pulling the oral side of the stump, the tissue around the left recurrent nerve was dissected. Then, the thoracic esophagus was completely mobilized circumferentially.

#### 2.2.2 Laparoscopic surgery

The patients were re-positioned to supine. The port positions were as follows (Fig. 3b): The laparoscopic camera port was in the umbilicus, and two operating ports were on each side of the abdominal wall; the other two ports were in the left side of the abdominal wall and below the xiphoid. The surgeon stood between the patient's legs. After isolation of the stomach laparoscopically, and a small incision was made below the xiphoid. Gastric tube reconstruction was performed through the retrosternal route.

An incision was made on the left side of the neck. A cervical, hand-sewn, end-to-side anastomosis in the esophagogastrostomy was performed. Albert-Lambert suture was used for posterior wall anastomosis, and Gambee suture was used for anterior wall anastomosis.

### 2.3 Result

**Fig. 5:**
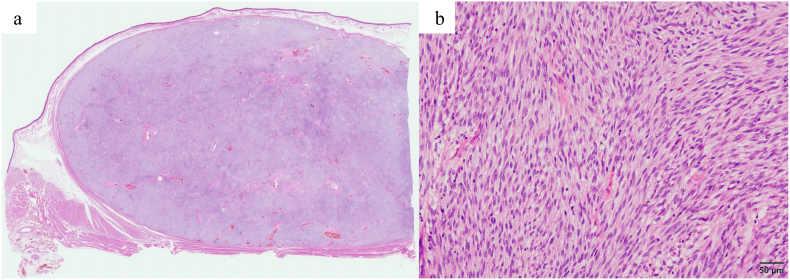
Pathological findings. (a) The tumor was 4.3 × 4.3 × 2.7 cm in size. (b) Spindle-shaped cells were growing in an intricate manner (magnification, ×200).

The duration of the thoracoscopic part was 69 min and the total operation time was 319 min. Total blood loss was 135 ml. There were no intraoperative complications. The patient's postoperative course was uneventful after surgery. On the first day after surgery, the patient started to take enteral nutrition and on the 7th day, she resumed eating. The patient was discharged 18th day after mainly due to social issues and no complications were observed. Macroscopically, the tumor was a well-circumscribed gray-white fibrous mass involving the submucosa and the muscular layers and sparing the mucosa of the esophagus, 4.3 × 4.3 × 2.7 cm in size (Fig. 5a). Histopathologically, spindle-shaped cells having a spindle-shaped nucleus and a pale eosinophilic vesicle, growing intricately manner, were observed (Fig. 5b). Immunohistochemical studies were performed and tumor cells stained diffusely for KIT, and CD34. None of the cells stained for pS100 or desmin. The proliferation index, using Ki-67, was 2–3%. The pathological diagnosis of the specimen confirmed low-risk GIST, so the patient was not given adjuvant therapy. We have been followed up every 3 months and the patient has had no recurrence 50 months after surgery.

## 3 Discussion

GISTs are reported as less than 1% of gastrointestinal malignancies and occur in the smooth muscle layer or mucosal muscle plate from the esophagus to the rectum [4]. Esophageal GISTs are said to be less than 1% of the total gastrointestinal GIST and relatively rare disease [1]. Additionally, the disease-specific, disease-free, and overall survival rates are significantly less in esophageal GIST compared to gastric GIST, likely secondary to the propensity for esophageal GIST to have higher mitotic rates and larger size at diagnosis [2].

CT scan and Magnetic resonance imaging, EUS are recommended for the diagnosis of GIST, but it is difficult to distinguish them from other submucosal tumors by imaging alone. EUS-FNA is the only useful diagnostic method, but the available facilities are limited and involve the risk of dissemination [5]. Generally, complete surgical resection is the standard of treatment for primary GIST because of malignant potential. However, which surgical procedure should be performed is still under debate due to the anatomical peculiarity of the esophagus, and the surgical options are limited to esophagectomy or tumor enucleation. In general, enucleation of esophageal GIST is recommended for smaller tumors (2 to 5 cm) [6], [7]. However, the prognosis of esophageal GIST is less favorable compared with gastric GIST [2], esophagectomy might be preferable for the goal to achieve R0 resection by highest radicality. It has been advocated that tumors greater than 2 cm be treated with esophagectomy, given the delicate nature of the tumor and the theoretical difficulty of enucleating a large tumor [6]. In the present case, McKeown or Ivor Lewis esophagectomy was an option because of the location and size of the tumor. In our department, McKeown's procedure in the prone position has been the first choice for esophageal cancer. From the viewpoint of postoperative anastomotic insufficiency, we think McKeown's procedure has some advantages compared to Ivor Lewis's procedure which needs intra-thoracic anastomosis.

Robotic surgical systems were developed to aid in overcoming the technical limitations of conventional minimally invasive surgery. Robotic surgery has several technological advantages as it provides a three-dimensional view, ten times magnification, tremor control, and ambidexterity. They compensate for the technical shortcomings of conventional laparoscopic or thoracoscopic surgery and allow for more intuitive laparoscopic or thoracoscopic surgery operations [8], [9]. RAMIE was introduced in 2003 and found to be a safe technique with good oncological outcomes in the first reported case series [10], [11], [12]. RAMIE was associated with less intraoperative blood loss, lower postoperative pain scores, faster functional recovery, and better quality of life when compared to open esophagectomy [13]. Theoretically, RAMIE seems to get better short-term and oncological results, which could be supported by the facilitated manipulation and precise dissection of the robotic system [11]. However, controversy still exists on the potential advantages of RAMIE versus minimally invasive esophagectomy (MIE). In recent years, there have been several reports comparing MIE and RAMIE for esophageal cancer. Y. Yang et al. reported that RAMIE will result in at least similar oncologic outcomes and long-term quality of life, but with a shorter operation time, a lower percentage of perioperative complications, lower blood loss, and shorter hospital stay when compared with MIE [14]. D. Jin et al. suggested that RAMIE and MIE mainly display similar effects and safety, but RAMIE could reduce the risk of nerve damage due to its good visual field and flexibility, and the intraoperative blood loss is less than MIE [15]. In addition, robotic assistance has been suggested to improve the surgeon's ergonomic conditions when operating [16]. With the development of robotic-assisted techniques and thoracic surgeons gaining experience, RAMIE might be beneficial for an increasing number of patients.

In recent years, imatinib has been widely used as adjuvant therapy. The current guidelines for all GIST from NCCN [17] recommend the us e of sunitinib, avapritinib, and regorafenib in addition to imatinib. However, since reports of the efficacy of adjuvant therapy for esophageal GIST are limited to case series or case reports, evaluation of its efficacy still needs to be addressed. More clinicopathological data and clinical trials involving esophageal GIST are expected.

## 4 Conclusion

Complete surgical resection is the standard of treatment for esophageal GIST because of malignant potential. But there is no standard technique due to the anatomical peculiarity of the esophagus. RAMIE may be useful for esophageal GIST because it facilitates safe and minimally invasive surgery in a limited space of the thoracic cavity.

## Consent

Written informed consent was obtained from the patient for the publication of this report and any accompanying images.

## Provenance and peer review

Not commissioned, externally peer-reviewed.

## Ethical approval

We obtained permission from the ethics committee in our institution.

## Funding

This research did not receive any specific grant from any funding agencies in the public, commercial, or not-for-profit sectors.

## CRediT authorship contribution statement

Hiroyuki Yamamoto: Conceptualization; Data curation; Investigation; Visualization; Writing-original draft.

Yuma Ebihara: Conceptualization; Methodology; Project administration; Writing-review & editing.

Kimitaka Tanaka, Aya Matsui, Yoshitsugu Nakanishi, Toshimichi Asano, Takehiro Noji, Yo Kurashima, Soichi Murakami, Toru Nakamura, Takahiro Tsuchikawa, Keisuke Okamura, Toshiaki Shichinohe: Conceptualization; Data curation; Writing-review & editing.

Satoshi Hirano: supervision.

All authors read and approved the final manuscript.

## Guarantor

Yuma Ebihara.

## Research registration number

We performed informed consent fully and got consent from the patient.

1. Name of the registry:
2. Unique identifying number or registration ID:
3. Hyperlink to your specific registration (must be publicly accessible and will be checked):

## Declaration of competing interest

This research did not receive any specific grant from funding agencies in the public, commercial, or not-for-profit sectors.
